# Novel Machine Learning Approaches Revolutionize Pancreatic Malignancy Prognosis: Exploring Programed Cell Death

**DOI:** 10.1155/mi/4068444

**Published:** 2025-10-31

**Authors:** Na Xu, Xiaye Miao, Jiali Jiang, Xue Han, Lirong Kuang, Tiantian Fan, Qing Zhang, Xiaoyan Wang

**Affiliations:** ^1^Department of Geriatrics, Zhangjiagang Hospital Affiliated to Soochow University, Suzhou, China; ^2^Department of Laboratory Medicine, Northern Jiangsu People's Hospital, Yangzhou, China; ^3^Department of Gastroenterology, Huai'an Second People's Hospital, The Affiliated Huai'an Hospital of Xuzhou Medical University and the Second People's Hospital of Huai'an, Huai'an, Jiangsu, China; ^4^Department of Ophthalmology, Wuhan Wuchang Hospital (Wuchang Hospital Affiliated to Wuhan University of Science and Technology), Wuhan, China; ^5^Department of Physical Examination, Huai'an Second People's Hospital, The Affiliated Huai'an Hospital of Xuzhou Medical University and the Second People's Hospital of Huai'an, Huai'an, Jiangsu, China; ^6^Department of Hepatology, Huai'an No.4 People's Hospital, Huai'an, Jiangsu, China; ^7^Department of Radiation Oncology, The Third Affiliated Hospital of Wenzhou Medical University, Wenzhou, China

**Keywords:** machine learning, pancreatic ductal adenocarcinoma, prognostication, programed cell demise, tumor microenvironment

## Abstract

Pancreatic ductal adenocarcinoma (PDAC) remains a highly aggressive malignancy with a poor prognosis and limited effective treatment options. Our study comprehensively explores the complex role of programed cell death (PCD) mechanisms in PDAC development, examining 18 distinct PCD pathways and their genetic underpinnings. Using an advanced machine learning framework incorporating 429 algorithmic variations, we have developed an innovative PCD-based molecular signature that demonstrates robust prognostic capabilities. This signature exhibits superior performance across diverse patient cohorts, significantly outperforming traditional clinicopathological indicators. Through integrated pathway analysis, we revealed that high-risk patients show distinct activation of oncogenic pathways and significant alterations in the tumor immune microenvironment. These alterations include reduced infiltration of cytotoxic T lymphocytes and increased levels of immunosuppressive regulatory T cells (Tregs). Furthermore, leveraging the TISCH (Tumor Immune Single Cell Hub) database, we conducted detailed single-cell expression profiling of our signature genes across different cell populations within the tumor microenvironment (TME). This analysis uncovered cell-type-specific expression patterns of key PCD-related genes. Our results highlight the critical involvement of PCD in PDAC progression and introduce a promising tool for clinical risk stratification. The integration of bulk and single-cell transcriptomic analyses not only validates our molecular signature but also reveals potential cellular targets for therapeutic intervention. This PCD-focused approach may support the development of personalized therapeutic strategies and ultimately improve outcomes for PDAC patients.

## 1. Introduction

Pancreatic ductal adenocarcinoma (PDAC) is an extremely aggressive malignancy, marked by a dismal 5-year survival rate of just 12% [1]. The incidence of PDAC has doubled over the last 20 years [2]. At present, surgical removal remains the only treatment option with curative potential [3]. However, the majority of patients present with advanced-stage disease, rendering them ineligible for operative management [4]. Even after complete surgical resection, the 5-year survival rate is a bleak 25%, largely attributable to the recurrence of the disease locally or the development of distant metastases [5]. This challenging clinical landscape highlights the urgent necessity for innovative prognostic biomarkers to improve patient prognosis [6]. Aberrant alterations in the tumor microenvironment (TME) have been shown to be closely associated with patient prognosis across multiple cancer types [7–12].

Cell death represents a fundamental biological process that orchestrates numerous physiological and pathological phenomena, ranging from development and aging to tumorigenesis [13, 14]. Based on the underlying molecular mechanisms, cell death can be broadly categorized into two major types: programed cell death (PCD) and accidental cell death (ACD). Unlike ACD, PCD is an intricately orchestrated and tightly regulated process governed by specific molecular pathways under precise genetic control, rendering it susceptible to both pharmacological and genetic interventions. A significant milestone was reached in 2018 when the Nomenclature Committee on Cell Death established comprehensive guidelines delineating the morphological and biological characteristics of cell death, identifying 12 distinct forms, including necroptosis and immunogenic cell death (ICD) [15]. The landscape of cell death mechanisms has since expanded considerably, with recent discoveries unveiling additional crucial modalities operating in diverse biological contexts. These newly characterized forms include autosis, cuproptosis, anoikis, disulfidptosis, alkaliptosis, oxeiptosis, and mitotic catastrophe, each contributing uniquely to cellular homeostasis and disease progression.

Cellular demise plays diverse and critical roles throughout the progression of cancer, with evasion of cell death being a defining characteristic of malignancy [16]. Its influence extends to the effectiveness and tolerance of various antineoplastic therapies, such as radiotherapy and immunotherapy. For example, high-energy ionizing radiation (IR) contributes to its antitumor activity in part by triggering ferroptosis, thereby increasing tumor sensitivity to radiation [17, 18]. Interestingly, ferroptosis can also foster an immunosuppressive TME by altering the migration and polarization of tumor-associated macrophages (TAMs), which may reduce the effectiveness of immune checkpoint blockade therapies [19]. However, the precise relationship between PCD and PDAC remains incompletely elucidated.

With the accumulation of large-scale genomic and clinical data, machine learning approaches, with their powerful capabilities in data analysis and pattern recognition, have emerged as ideal tools for constructing predictive models [20–25]. This investigation represents the first comprehensive evaluation of PCD dynamics in PDAC, integrating 18 distinct PCD modalities and multiple PCD-associated genetic determinants. Through an in-depth analysis of large-scale genomic and clinical datasets, it introduces a novel metric for quantifying tumoral PCD, termed the PCD signature, and establishes a robust correlation between PCD dynamics and patient prognosis.

## 2. Methods

### 2.1. Cohort Assembly and Data Harmonization

This investigation represents the first comprehensive analysis of PCD dynamics in pancreatic PDAC, incorporating 18 distinct PCD modalities and their associated genetic determinants. Through rigorous analysis of extensive genomic and clinical datasets, we developed and validated a novel metric—the PCD signature—for quantifying tumor-specific PCD activity and its correlation with patient outcomes. Our study utilized a rigorously curated dataset comprising 1165 PDAC specimens collected from eight independent cohorts: TCGA-PAAD (*n* = 177), ICGC-PDAC-AU (*n* = 267), ICGC-PDAC-CA (*n* = 184), E-MTAB-6134 (*n* = 288), GSE62452 (*n* = 65), GSE28735 (*n* = 42), GSE85916 (*n* = 79), and GSE57495 (*n* = 63). Strict inclusion criteria were implemented, mandating a minimum cohort size of 40 cases with complete survival data, histopathologically confirmed PDAC diagnosis, and treatment-naïve primary neoplasms at surgical intervention. For comparative analyses, we incorporated 167 non-neoplastic pancreatic tissue samples from the Genotype-Tissue Expression (GTEx) project, accessed through UCSC Xena. Data preprocessing followed a standardized protocol to ensure cross-platform compatibility and minimize technical variation. Raw sequencing data from TCGA and GTEx underwent normalization to transcripts per kilobase million (TPM), while preprocessed expression matrices from ArrayExpress, GEO, and ICGC were utilized as provided by their respective repositories. To establish uniformity, we applied log_2_ transformation across all datasets. Notably, to mitigate batch effects inherent in multicohort analyses, we employed the ComBat algorithm from the “sva” package in R [26, 27], facilitating robust comparative analyses across these diverse PDAC datasets.

### 2.2. Differential Expression Analysis and Functional Characterization

To explore the molecular framework of PCD in PDAC, we performed a detailed transcriptomic analysis focused on PCD-associated genes curated from a pivotal study [28]. Using the “limma” package in R, we identified differentially expressed PCD-related genes (DEPRGs) by comparing PDAC tissues to non-neoplastic pancreatic samples, applying stringent thresholds (*p*.adjust < 0.05 and |log_2_FC| ≥ 1). Following this, we conducted Kyoto Encyclopedia of Genes and Genomes (KEGG) pathway and Gene Ontology (GO) enrichment analyses using the “clusterProfiler” R package [29]. These analyses revealed key biological pathways, molecular functions, and cellular components associated with dysregulated PCD processes. This integrated approach provides a systems-level understanding of the potential mechanisms driving PDAC development and progression, offering valuable insights into its molecular pathogenesis.

### 2.3. Construction of a Prognostic PCD Signature via Advanced Machine Learning

To develop a high-accuracy prognostic model for PDAC based on PCD markers, we employed an advanced machine learning framework. This framework incorporated 27 algorithmic approaches, spanning classical statistical methods, modern machine learning techniques, and cutting-edge deep learning architectures. A total of 429 algorithmic permutations were explored [30, 31], including survival-specific models (e.g., CoxTime and DeepSurv), ensemble-based methods (e.g., random survival forests and gradient boosting machines), and regularized regression techniques (e.g., LASSO and Ridge). Feature selection began with univariate Cox regression analysis of the TCGA cohort, identifying PCD-related genes significantly associated with prognosis (*p*  < 0.05). These features were then evaluated across the algorithmic suite to construct predictive models within the TCGA dataset. Model performance was thoroughly evaluated using concordance indices (*C*-indices) across multiple independent validation cohorts. The final prognostic signature was selected based on the highest mean *C*-index, ensuring robust predictive accuracy and strong generalizability to external datasets.

### 2.4. Molecular Pathway Characterization of the PCD Signature

To unravel the biological basis of the PCD signature, we carried out an extensive functional annotation using a combination of analytical techniques. Gene Set Variation Analysis (GSVA) and Gene Set Enrichment Analysis (GSEA) were employed, utilizing the rich resources of the Molecular Signatures Database (MSigDB). These methods allowed us to identify key molecular pathways, biological processes, and functional gene sets associated with the PCD signature, providing valuable insights into its role in pancreatic adenocarcinoma [32]. These analyses, conducted using the “GSVA” and “clusterProfiler” R packages [29, 33] revealed key pathways and biological processes enriched in the PCD signature. To validate and extend these findings, we utilized Metascape [34], an advanced integrative platform for gene annotation and pathway analysis. This comprehensive, multipronged approach provided a detailed examination of the molecular mechanisms and functional roles underpinning the prognostic utility of the PCD-based stratification in pancreatic adenocarcinoma, offering deeper insights into its biological significance.

### 2.5. Characterization of Tumor Immune Microenvironment

To comprehensively map the immune landscape associated with the PCD signature, we adopted a multidimensional analytical strategy. The CIBERSORT algorithm, a robust deconvolution tool, was used to estimate the relative proportions of 22 immune cell types within bulk tumor transcriptomic data [21, 35]. This was further enriched by incorporating immune infiltration metrics from the pan-cancer immune profiling study conducted by Thorsson et al. [36]. Furthermore, we assessed the expression of 29 well-established immune-related gene signatures, as described by He et al. [37], using GSVA [33]. To gauge cytolytic (CYT) potential, we computed a CYT activity score based on the geometric mean expression of granzyme A (GZMA) and perforin 1 (PRF1) [38]. Together, these complementary analyses provided a detailed characterization of the tumor immune microenvironment, offering insights into the interplay between PCD and immune dynamics in pancreatic adenocarcinoma.

### 2.6. Single-Cell Expression Analysis

We retrieved single-cell RNA sequencing (scRNA-seq) of PDAC from a previously published investigation [39]. Rigorous filtering of the gene-cell matrix was performed, excluding cells with fewer than 1000 or more than 1000 features, and mitochondrial gene content exceeding 15%. Following stringent quality control, 122,074 high-quality cells were selected and subsequently processed using the Seurat R package for comprehensive analysis [40]. Gene expression normalization was accomplished through the LogNormalize algorithm, applying a standardization factor of 10,000. Subsequently, we identified and extracted the top 2000 most variable genes, followed by expression level scaling and principal component analysis (PCA) within the variable gene space. To mitigate potential batch effects, we implemented the Harmony R package [41]. All computational procedures were executed utilizing integrated functions from Seurat and Harmony packages, encompassing critical steps such as data normalization, variable feature identification, scaling, dimensionality reduction, neighborhood construction, clustering, and uniform manifold approximation and projection (UMAP) visualization.

### 2.7. Cell–Cell Communication

We comprehensively integrated gene expression data and systematically evaluated intercellular communication networks using the CellChat computational framework [42, 43]. Employing the predefined CellChatDB ligand-receptor database, we rigorously followed the established CellChat analytical protocol. Through meticulous identification of differentially overexpressed ligands and receptors across distinct cellular populations, we precisely inferred cell type-specific molecular interactions. Our approach enabled the detection of significantly enhanced ligand-receptor communication pathways where either signaling molecules or their corresponding receptors demonstrated substantially elevated expression levels.

### 2.8. qRT-PCR

Cell lines were obtained from the Shanghai Institutes for Life Sciences, Chinese Academy of Sciences, Shanghai, China. The pancreas epithelial cell line HPNE as well as the pancreatic cancer cell lines SW1990 were cultured in RPMI 1640 medium supplemented with 10% fetal bovine serum, 10 U/mL penicillin, and 50 µg/mL streptomycin, maintained at 37°C in an atmosphere with 5% carbon dioxide. Total RNA was isolated from cellular and tissue samples using Trizol reagent (Invitrogen), and reverse transcription was carried out with SuperScript II reverse transcriptase (Invitrogen) in accordance with the manufacturer's instructions.

### 2.9. Statistical Analysis

All statistical analyses and data visualizations were executed using R version 4.3.1. Continuous variables underwent comparative analysis using either the Wilcoxon rank-sum test or Student's *t*-test, depending on data distribution characteristics. To stratify patients based on our PCD signature, optimal cut-off values were determined utilizing the “survminer” package. Survival analyses were conducted employing Kaplan–Meier estimators, implemented through the “survival” R package. The discriminative ability of our prognostic model was assessed via *C*-indices, calculated using the “survival” package. Time-dependent receiver operating characteristic (ROC) curves and corresponding area under the curve (AUC) values were generated using the “timeROC” package, providing dynamic assessment of predictive accuracy. Statistical significance was consistently defined as *p*  < 0.05, unless explicitly stated otherwise for specific analyses.

## 3. Results

### 3.1. Molecular Profiling of PCD-Associated Genes in PDAC

**Figure 1:**
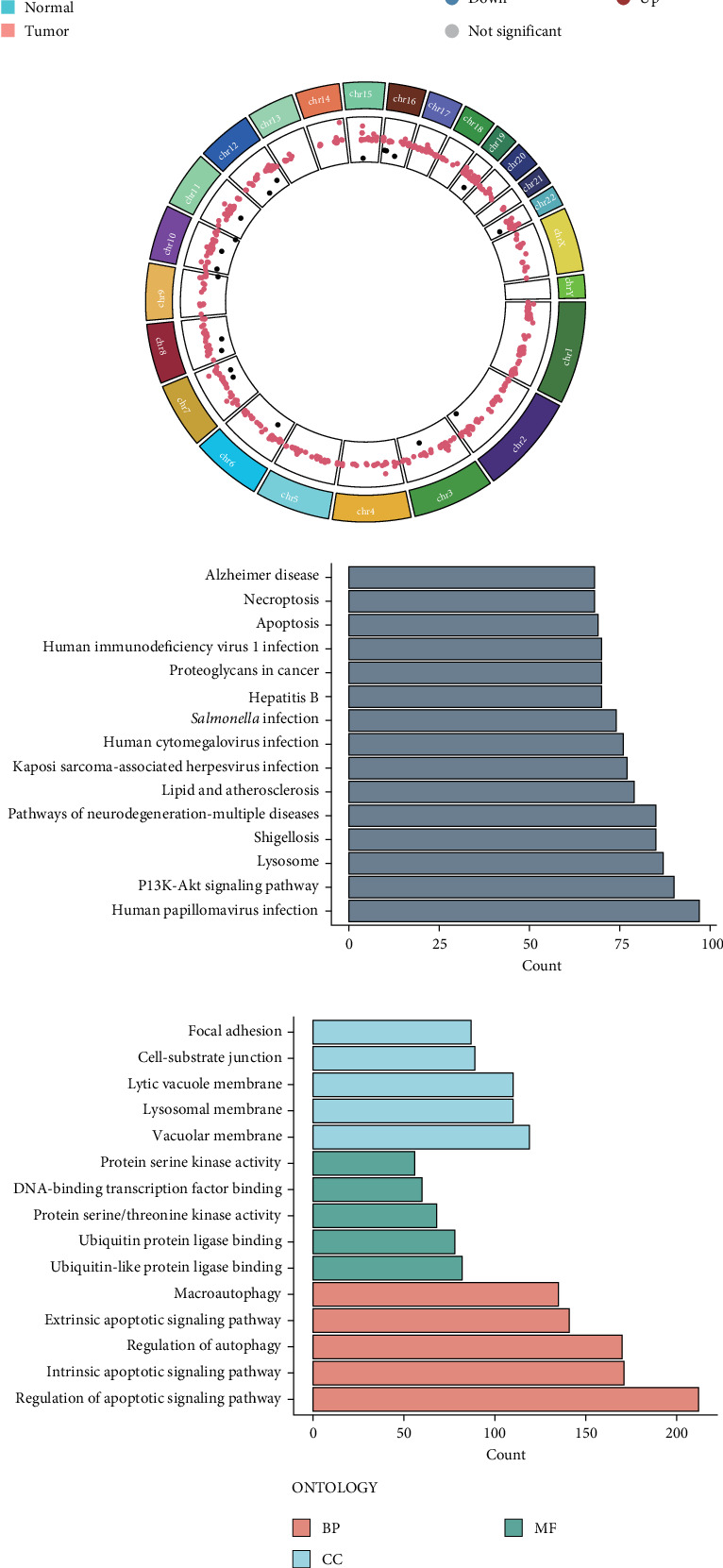
Comprehensive molecular profiling of PCD-associated gene expression patterns in PDAC. (A) Unsupervised hierarchical clustering analysis and heat map visualization of DEPRGs comparing PDAC specimens with adjacent non-neoplastic pancreatic tissue. (B) Volcano plot analysis demonstrating the distribution of DEPRGs; downregulated genes depicted in blue, upregulated genes in red, and nonsignificantly altered genes in gray. Significantly dysregulated DEPRGs (adjusted *p*  < 0.05, |log2FC| > 1) are annotated. (C) Genome-wide chromosomal distribution analysis of DEPRGs identified in the TCGA cohort. (D) KEGG pathway enrichment analysis revealing significantly enriched biological pathways associated with DEPRGs. (E) Gene Ontology (GO) functional annotation analysis demonstrating enriched biological processes, molecular functions, and cellular components associated with DEPRGs. (F) Comprehensive genomic alteration landscape of PCD-related genes across TCGA PDAC specimens visualized through oncoplot representation.

Comprehensive analysis of the TCGA-PDAC cohort unveiled extensive dysregulation of PCD-related genes in PDAC. Applying stringent statistical criteria (adjusted *p*  < 0.05, |log_2_FC| > 1), we identified 917 DEPRGs, with a striking predominance of upregulated genes (893 genes) compared to downregulated ones (24 genes) in tumor tissues relative to nonmalignant pancreatic samples. To visualize these molecular alterations comprehensively, we generated multiple graphical representations: heatmaps depicting scaled RNA expression patterns, volcano plots illustrating fold changes and statistical significance, and chromosomal distribution maps of DEPRGs (Figure 1). In-depth functional characterization through KEGG and GO enrichment analyses revealed significant enrichment of these DEPRGs in crucial oncogenic pathways, particularly PI3K-Akt signaling, cancer-associated proteoglycans, and focal adhesion mechanisms (Figure 1D,E). Notably, our mutational landscape analysis demonstrated that a substantial proportion (80.74%) of PDAC patients harbored alterations in PCD-related genes. KRAS mutations emerged as the predominant genetic alteration, present in 71% of cases, followed by mutations in 19 other genes with varying frequencies ranging from 2% to 66% (Figure 1F). These comprehensive findings highlight the fundamental importance of PCD-related gene dysregulation and mutation in driving PDAC pathogenesis.

### 3.2. Development and Validation of a Prognostic PCD Signature in PDAC

**Figure 2:**
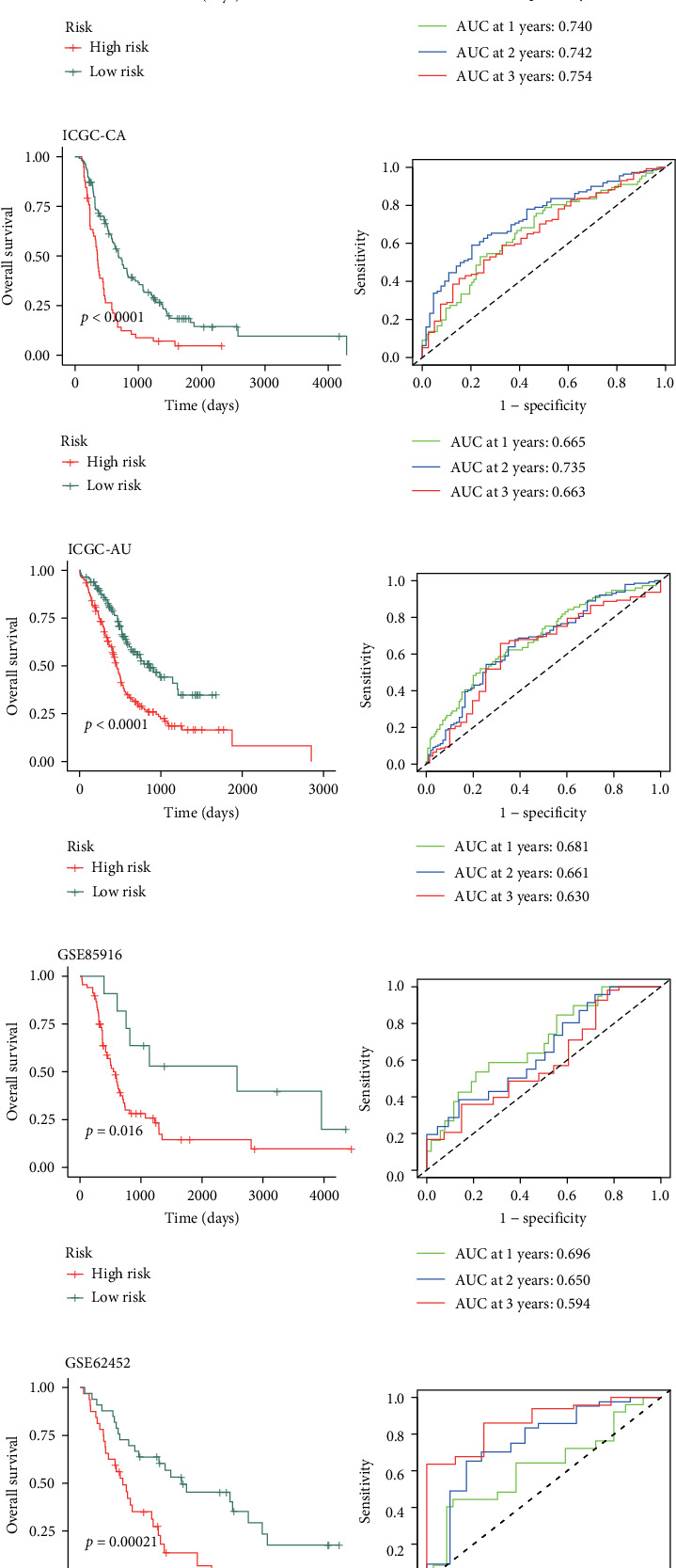
Generation and multicohort validation of an artificial intelligence-derived PCD prognostic signature. (A) Systematic performance assessment of 429 machine learning-derived prediction models utilizing 10-fold cross-validation methodology; concordance index (*C*-index) distribution across multiple independent datasets. (B–I) Stratification analysis of overall survival (OS) utilizing Kaplan–Meier survival estimation and time-dependent receiver operating characteristic (ROC) curves based on the PCD signature across multiple independent cohorts. (B) The Cancer Genome Atlas (TCGA), (C) International Cancer Genome Consortium-Canadian (ICGC-CA), (D) International Cancer Genome Consortium-Australian (ICGC-AU), (E) GSE85916, (F) GSE62452, (G) GSE57495, (H) GSE28735, and (I) E-MTAB-6134 patient populations.

From the TCGA-PDAC cohort, we identified 97 highly significant DEPRGs (*p*  < 0.001). To construct a reliable prognostic model, we systematically evaluated 429 algorithmic combinations, benchmarking their performance using *C*-indices across multiple validation datasets. The combination of CoxBoost and Ridge regression emerged as the most effective approach, achieving the highest mean *C*-index of 0.626 (Figure 2A). This optimized PCD signature enabled the stratification of PDAC patients into high- and low-risk groups based on an established threshold. Kaplan–Meier survival analyses consistently demonstrated significantly poorer overall survival (OS) among high-risk patients across all cohorts analyzed (*p*  < 0.05) (Figure 2). The model's prognostic accuracy and stability over time were validated using time-dependent ROC curves, which confirmed its robust performance in diverse PDAC populations (Figure 2). These findings highlight the utility of the PCD-based model for risk stratification and prognosis in PDAC.

### 3.3. Comprehensive Assessment of the PCD Signature's Prognostic Efficacy

**Figure 3:**
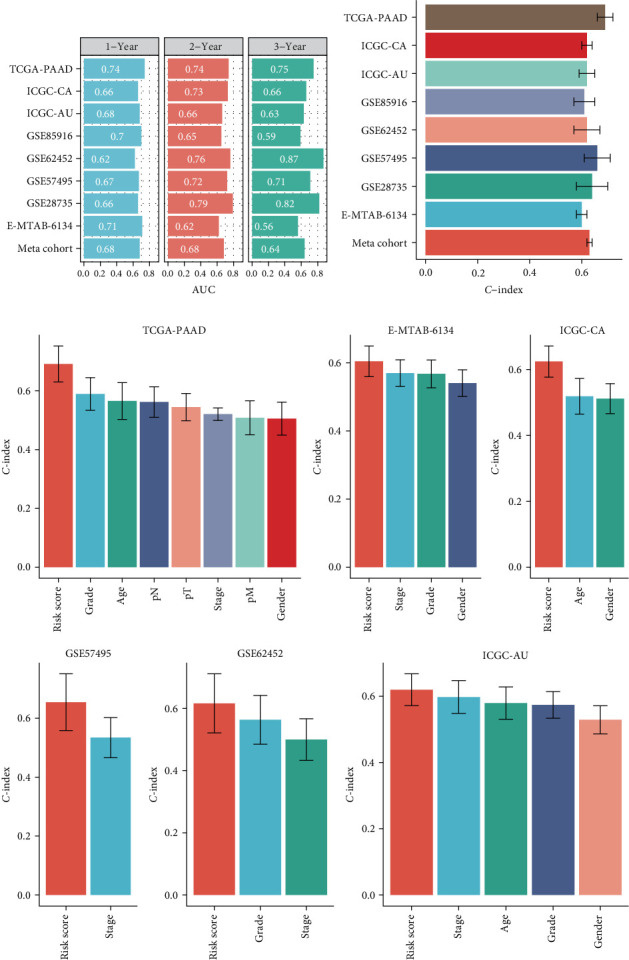
Comprehensive assessment of PCD signature prognostic performance. (A) Time-dependent ROC analysis for 1-, 2-, and 3-year OS prediction across cohorts. (B) Forest plot of *C*-indices with 95% confidence intervals for PCD signature across datasets. (C) Comparative analysis of PCD signature versus conventional clinicopathological parameters for prognostic accuracy in multiple PDAC cohorts.

To robustly assess the prognostic performance of our PCD signature, we conducted extensive ROC analyses across multiple PDAC cohorts. The AUC values for 1-, 2-, and 3-year survival predictions demonstrated consistent predictive ability, ranging from 0.56 to 0.87 across diverse datasets (Figure 3A). *C*-indices, along with 95% confidence intervals, were calculated for each cohort, confirming the signature's strong discriminative power (Figure 2B). A meta-cohort analysis further validated the PCD signature, yielding a pooled *C*-index of 0.63 (0.61–0.65), emphasizing its robustness and generalizability. Comparative assessments against traditional clinicopathological factors, such as TNM staging and histological grade, revealed the superior prognostic accuracy of the PCD signature (Figure 3C). These comprehensive analyses collectively establish the PCD signature as a highly reliable and clinically relevant tool for stratifying PDAC patients, outperforming conventional prognostic indicators in predicting OS outcomes. The mRNA expression of the key PCD signature gene MET was verified by qRT-PCR (Figure S1).

### 3.4. Biological Mechanisms Underpinning the PCD Signature

**Figure 4:**
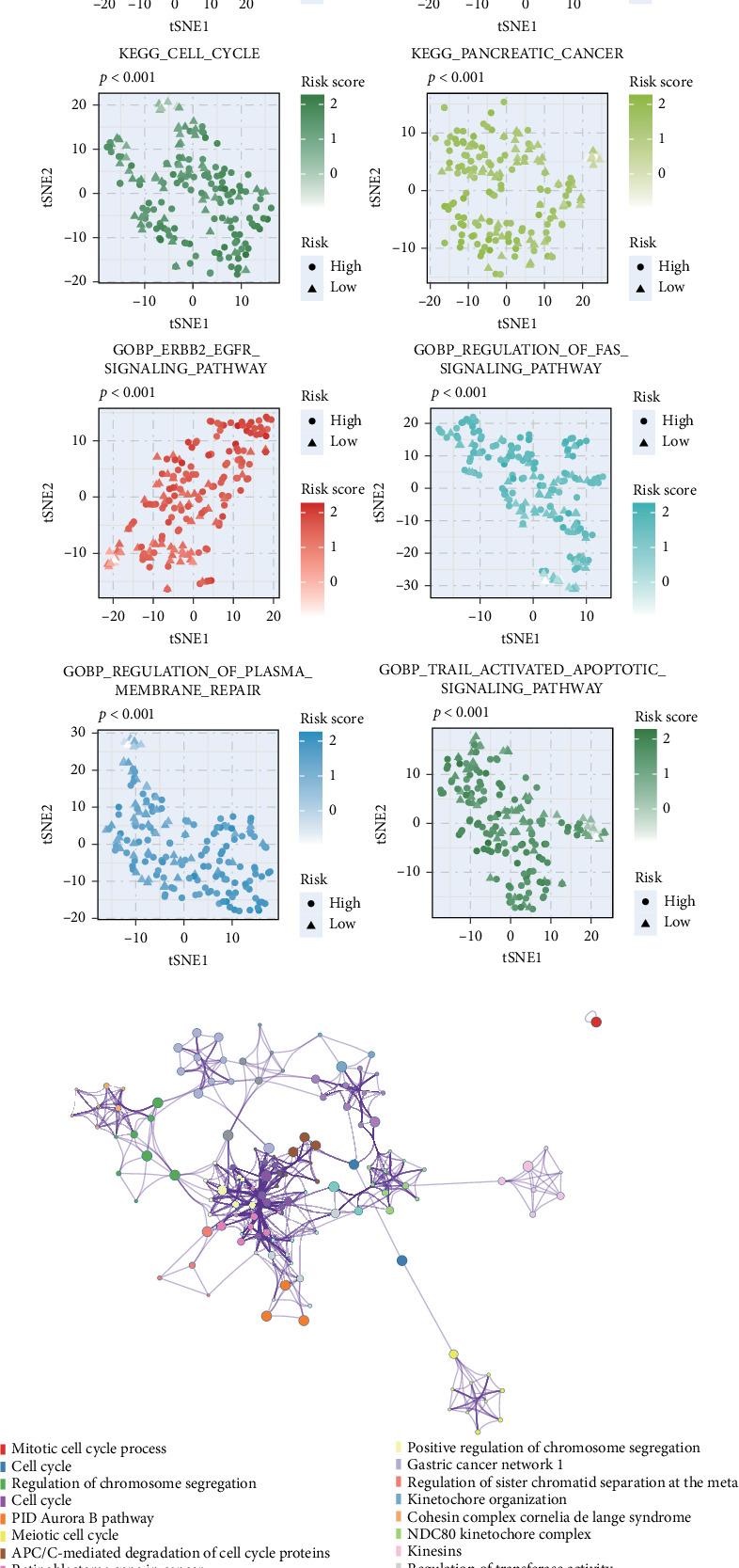
Functional annotation and pathway analysis of PCD signature-defined risk groups. (A) GSVA-derived heatmap illustrating differential pathway activities between high- and low-risk groups using MSigDB gene sets. (B) t-SNE visualization of KEGG and reactome pathway activities in risk-stratified groups. (C) Metascape-generated enrichment network of differentially expressed genes between risk groups. (D) GSEA plots highlighting significantly enriched KEGG pathways associated with PCD signature. *⁣*^*∗∗∗*^*p*  < 0.001.

To uncover the biological mechanisms linked to our PCD signature, we conducted extensive pathway analyses. Patients classified as high-risk showed significant enrichment in key oncogenic pathways, including glycolysis, mTORC1 signaling, and apoptotic cascades (*p*  < 0.05) (Figure 4A). Comparative analyses between risk groups highlighted pronounced differences in the activation of tumorigenic pathways (*p*  < 0.05) (Figure 4B). Further validation through GSEA and ORA confirmed the preferential enrichment of tumor-promoting pathways in the high-risk cohort (Figure 4C,D). These complementary approaches consistently indicated that the poor prognosis observed in high-risk patients is likely driven by the upregulation of these oncogenic signaling networks. These findings provide crucial mechanistic insights into the prognostic efficacy of the PCD-based stratification in PDAC.

### 3.5. Immunological Profiling Associated With PCD-Based Risk Stratification in PDAC

**Figure 5:**
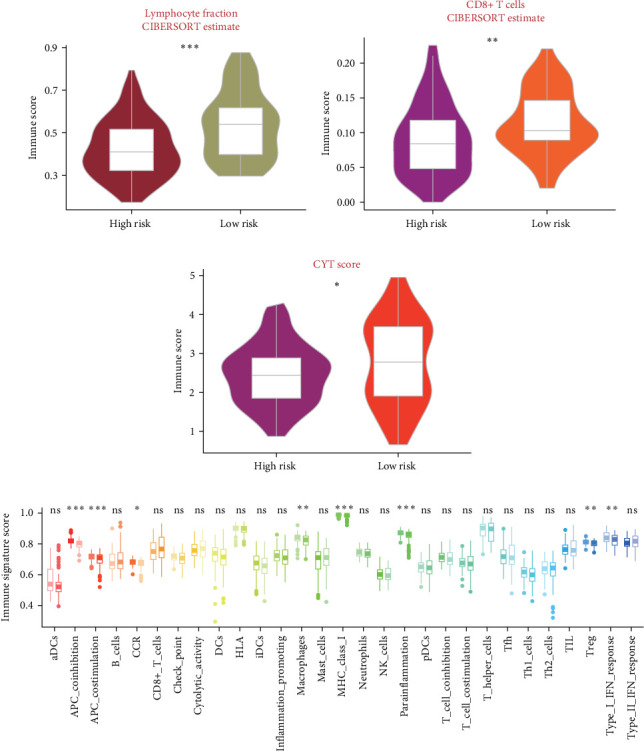
Immunological landscape associated with PCD signature in PDAC. (A) Comparative analysis of lymphocyte fractions (CIBERSORT-derived) between risk groups. (B) Tumor-infiltrating lymphocyte (TIL) composition differences between high- and low-risk patients. (C) CD8+ T cell abundance comparison across risk groups. (D) Boxplot array depicting 29 immune-related gene signatures (ssGSEA-derived) stratified by PCD risk groups, highlighting differential immune microenvironment characteristics. ns *p* > 0.05, *⁣*^*∗*^*p* < 0.05, *⁣*^*∗∗*^*p* < 0.01, and *⁣*^*∗∗∗*^*p* < 0.001.

Our analysis of the tumor immune microenvironment revealed striking differences between the PCD signature-defined risk groups in PDAC. High-risk patients showed a significant depletion of lymphocyte populations compared to low-risk individuals (*p*  < 0.05) (Figure 5A). This depletion was particularly evident in immune-stimulatory cell subsets, notably CD8+ T cells, which were markedly reduced in the high-risk cohort (*p*  < 0.05) (Figure 5B). These observations were corroborated by alternative immune quantification methods, including immune signature scores, which consistently confirmed diminished CD8+ T cell abundance in high-risk tumors (Figure 5D). CYT activity scores, which quantify CD8+ T cell functionality, were markedly higher in the low-risk group, reflecting greater cytotoxic capacity and immune activity [38] (Figure 5C). Conversely, regulatory T cell (Treg) infiltration was significantly higher in high-risk tumors, indicating a more immunosuppressive microenvironment that could impair antitumor immune responses [44, 45] (Figure 5D).

### 3.6. Detailed Exploration of PCD Signature Distribution and Cellular Interactions in Different Cell Types

**Figure 6:**
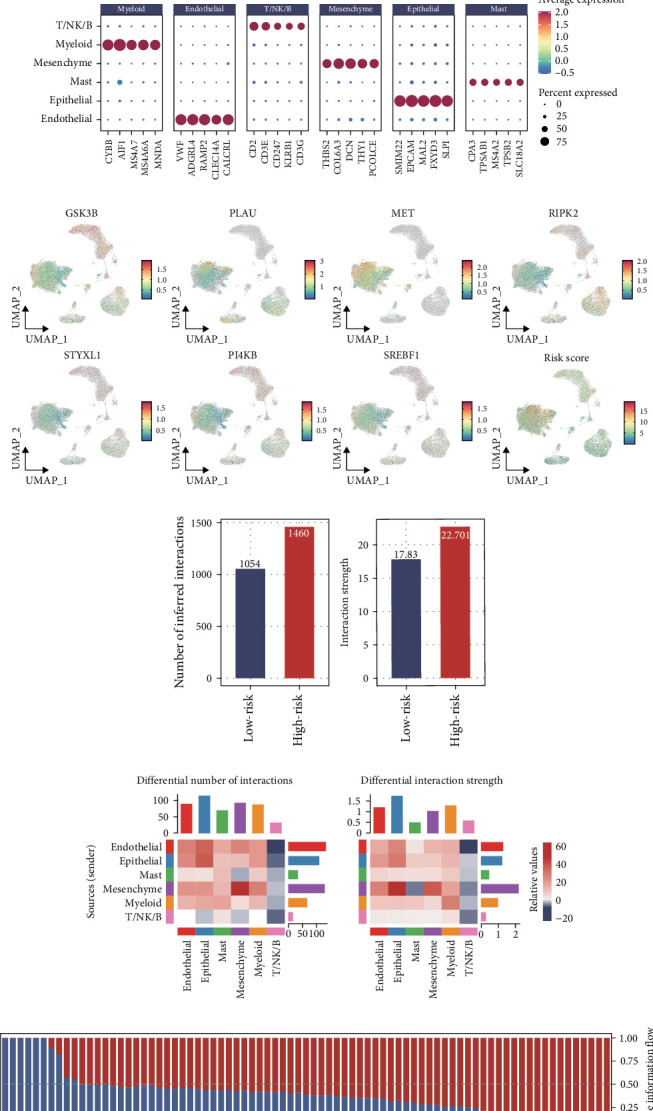
Detailed exploration of PCD signature distribution and cellular interactions in different cell types. (A,B) PDAC scRNA-seq data clustering and cell type annotations. (C) Abundance patterns of PCD signature genes (GSK3B, MET, PI4KB, PLAU, RIPK2, and STYXL1) and PCD signature scores across different cell types in PDAC. (D) Comparison of total number of cell–cell interactions and interaction strength between high and low risk groups. (E) Heatmaps showing differential number of interactions and interaction strength between cell types in the tumor microenvironment. (F) Relative signaling pathway activities between high and low risk groups, with red indicating enhanced activity in high risk group and blue indicating enhanced activity in low risk group.

Figure 6A,B illustrate PDAC clustering and cell type annotations, respectively. Key genes within the PCD signature and their abundance are presented in Figure 6C, revealing predominant expression in epithelial cells. By stratifying cells into high and low risk groups using the median score, we conducted cellular communication analysis. Notably, the high risk group demonstrated significantly enhanced intercellular interactions (Figure 6D,E). Furthermore, the high risk group was primarily enriched in GRN, TWEAK, and KIT tumor-related signaling pathways (Figure 6F). Thus, our study reveals that elevated PCD signature scores correlate with enhanced cellular interactions.

## 4. Discussion

PCD encompasses a wide range of genetically programed mechanisms of cellular self-destruction, playing critical roles in embryogenesis, tissue homeostasis, and immune regulation. This includes well-studied pathways such as apoptosis, necrosis, autophagy, and pyroptosis [15]. Despite its importance, a comprehensive analysis of PCD dynamics and their molecular characteristics in PDAC has been lacking. In this study, we employed advanced machine learning algorithms to construct a novel PCD-based prognostic signature for PDAC patients. Using a rigorous computational approach, we identified an optimal hybrid model combining Coxboost and Ridge regression, which exhibited strong predictive performance across multiple validation cohorts. ROC analyses demonstrated the signature's high discriminative power for predicting 1-, 2-, and 3-year OS. Importantly, the PCD signature consistently outperformed traditional clinicopathological factors, such as TNM staging and histological grade, in prognostic accuracy. These results highlight the clinical potential and translational relevance of our PCD-based prognostic tool for improving PDAC management.

The existing prognostic framework for PDAC is largely centered around TNM staging and the presence of metastasis [46]. However, the rise of PCD-related gene expression profiling signals a shift toward more advanced molecular prognostic approaches. The development of high-throughput RNA sequencing technologies has empowered clinical laboratories to uncover transcriptomic signatures with significant prognostic relevance [47]. Our study presents a cutting-edge artificial intelligence framework for constructing an PCD-based prognostic signature in PDAC. This sophisticated computational platform incorporates 27 diverse algorithms, encompassing traditional regression techniques, machine learning approaches, and deep learning methodologies, achieving superior predictive accuracy compared to conventional prognostic models. By leveraging AI-driven feature selection, model optimization, and enhanced cross-cohort generalizability, this framework has produced a robust and clinically viable tool for stratifying PDAC patients based on prognosis.

ICD within the TME is crucial for initiating and shaping antitumor immune responses. This process involves the presentation of tumor antigens by dendritic cells to T lymphocytes, ultimately leading to tumor cell destruction and the development of long-lasting anticancer immunity [48]. Induction of alternative cell death modalities, such as necroptosis, ferroptosis, or pyroptosis, has been shown to enhance immune-mediated tumor suppression and inhibit metastatic progression [48–50]. These findings highlight the complex interplay between TME dynamics and PCD pathways. In PDAC, T cell infiltration patterns exhibit considerable heterogeneity [51]. Higher densities of CD8+ T cells or tumor-infiltrating lymphocytes (TILs) have been associated with better clinical outcomes in PDAC [52, 53]. In contrast, Tregs, a prominent immunosuppressive component within the pancreatic TME, contribute to tumor progression by directly supporting malignant cells and suppressing effector immune responses [54]. Our analysis demonstrated that high-risk PDAC patients, as identified by our PCD signature, had significantly lower fractions of CD8+ T cells and increased Treg infiltration compared to low-risk patients. Additionally, high-risk tumors exhibited a global depletion of lymphocyte populations. We found through single-cell sequencing analysis that the high-risk group was primarily enriched in GRN, TWEAK, and KIT tumor-related signaling pathways. GRN is involved in promoting tumor growth and angiogenesis. For example, in retinoblastoma, TAMs express GRN, which can increase their angiogenic potential and promote tumor progression [55]. TWEAK signaling, particularly in triple-negative breast cancer (TNBC), has been shown to drive cancer cell invasion and proliferation. TWEAK-overexpressing TNBC cells display enhanced tumor growth and metastasis [43]. These results suggest that low-risk PDAC tumors may represent “immunologically hot” environments, shedding light on the prognostic value of our PCD-based stratification system.

Despite these valuable findings, several limitations must be acknowledged. First, the retrospective design of our study highlights the need for prospective validation in larger and independent cohorts to ensure the robustness of our conclusions. Second, the presence of incomplete clinical data for certain patients may introduce analytical biases, potentially affecting the reliability of the results. Last, further in vitro and in vivo studies are crucial to clarify the specific biological roles of the PCD-related genes included in our signature and their contributions to PDAC pathogenesis and progression.

## 5. Conclusion

Our study introduces a novel prognostic tool for PDAC based on the expression patterns of PCD-related genes. This artificial intelligence-derived signature outperforms traditional clinicopathological parameters in predictive accuracy, offering improved capabilities for patient stratification. The consistent performance of the PCD-based model across multiple independent cohorts highlights its potential clinical utility in supporting personalized treatment strategies for PDAC patients. Additionally, our findings shed light on the complex interplay between PCD mechanisms and tumor immunobiology, providing valuable insights into PDAC progression. While these results are encouraging, prospective validation in larger and more diverse patient populations is crucial to confirm the signature's generalizability and clinical relevance. Future studies should aim to unravel the functional roles of the key PCD-related genes within the signature, which may reveal novel therapeutic targets for tackling this challenging malignancy.

## Data Availability

The comprehensive genomic and transcriptomic data utilized in this investigation are publicly accessible through the following repositories: 1. The Cancer Genome Atlas (TCGA): http://cancergenome.nih.gov/ 2. ArrayExpress: https://www.ebi.ac.uk/arrayexpress/ 3. Gene Expression Omnibus (GEO): https://www.ncbi.nlm.nih.gov/geo/. These curated databases contain all relevant datasets employed in our analyses.

## Supporting Information

Supporting Information Figure S1. The mRNA expression of the key PCD signature gene MET was verified by qRT-PCR.
