# Supplementary figures and images for: Novel Machine Learning Approaches Revolutionize Pancreatic Malignancy Prognosis: Exploring Programed Cell Death
Source: Mediators Inflamm. 2025 Oct 31;2025:4068444. doi: 10.1155/mi/4068444 (PMC12595226; doi:10.1155/mi/4068444)

**Relative mRNA expression of MET**

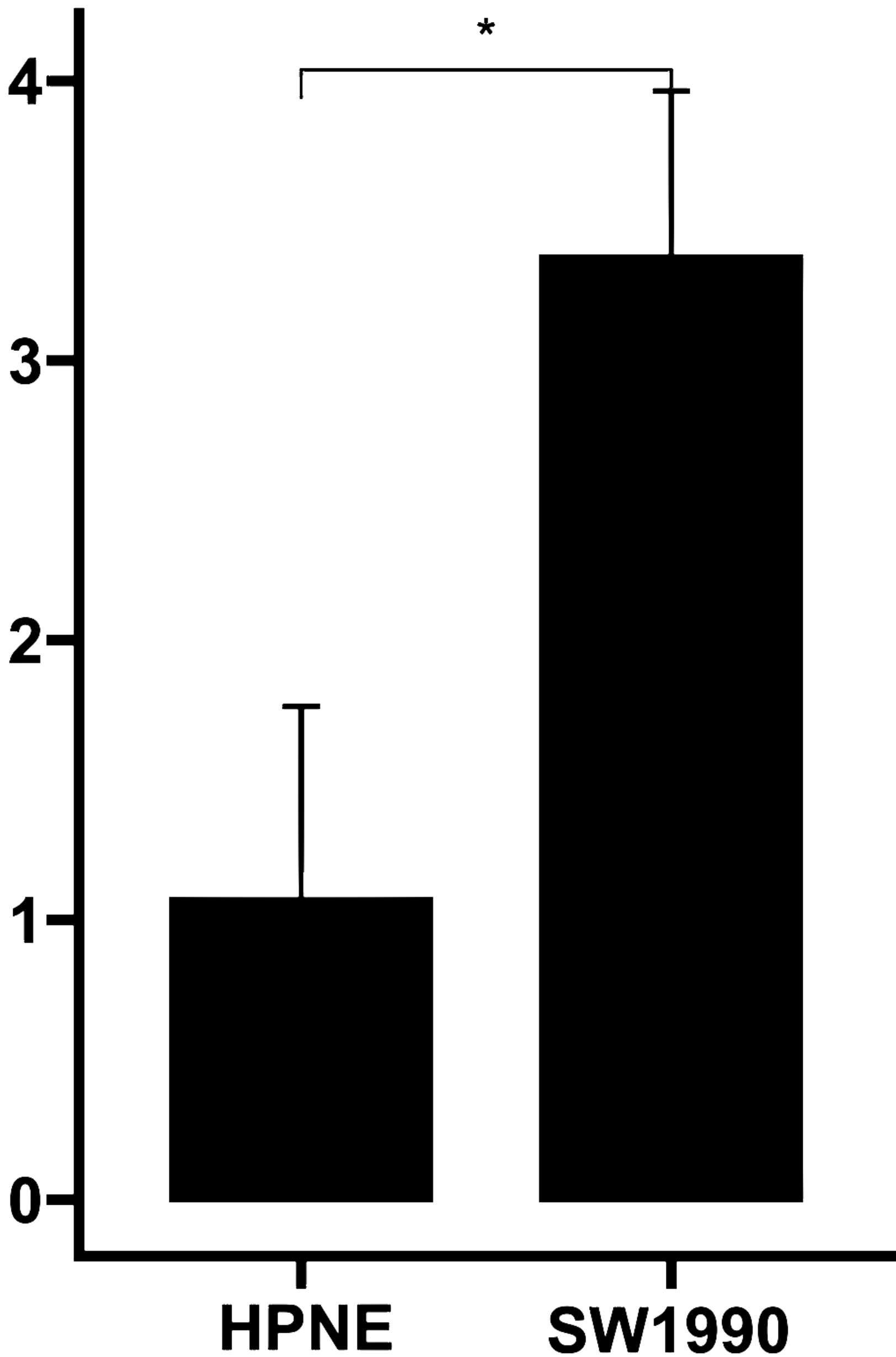

Supplement: Supporting Information — Figure S1. The mRNA expression of the key PCD signature gene MET was verified by qRT-PCR. [file 4068444.f1.pdf]
